# Neuroimaging-based analysis of DBS outcomes in pediatric dystonia: Insights from the GEPESTIM registry

**DOI:** 10.1016/j.nicl.2023.103449

**Published:** 2023-06-10

**Authors:** Bassam Al-Fatly, Sabina J. Giesler, Simon Oxenford, Ningfei Li, Till A. Dembek, Johannes Achtzehn, Patricia Krause, Veerle Visser-Vandewalle, Joachim K. Krauss, Joachim Runge, Vera Tadic, Tobias Bäumer, Alfons Schnitzler, Jan Vesper, Jochen Wirths, Lars Timmermann, Andrea A. Kühn, Anne Koy, on behalf of the GEPESTIM consortium

**Affiliations:** a Charité-Universitätsmedizin Berlin, Corporate Member of Freie Universität Berlin and Humboldt-Universität zu Berlin, Department of Neurology, Berlin, Germany; b Department of Pediatrics, Faculty of Medicine and University Hospital Cologne, University of Cologne, Cologne, Germany; c Department of Neurology, Faculty of Medicine, University of Cologne, Cologne, Germany; d Department of Stereotactic and Functional Neurosurgery, Faculty of Medicine and University Hospital Cologne, University of Cologne, Cologne, Germany; e Department of Neurosurgery, Hannover Medical School, Hannover, Germany; f Department of Neurology, University Medical Center Schleswig Holstein, Lübeck Campus, Lübeck, Germany; g Institute of System Motor Science, University Medical Center Schleswig Holstein, Lübeck Campus, Lübeck, Germany; h Department of Neurology, Institute of Clinical Neuroscience and Medical Psychology, Medical Faculty, Heinrich Heine University Düsseldorf, Düsseldorf, Germany; i Department of Neurology, Medical Faculty, Heinrich Heine University Düsseldorf, Düsseldorf, Germany; j Department of Neurology, University Hospital of Marburg, Marburg, Germany; k Center for Rare Diseases, Faculty of Medicine and University Hospital Cologne, University of Cologne, Cologne, Germany

**Keywords:** Dystonia, Pediatric, Deep brain stimulation, Connectomics, Sweetspot

## Abstract

**Introduction:**

Deep brain stimulation (DBS) is an established treatment in patients of various ages with pharmaco-resistant neurological disorders. Surgical targeting and postoperative programming of DBS depend on the spatial location of the stimulating electrodes in relation to the surrounding anatomical structures, and on electrode connectivity to a specific distribution pattern within brain networks. Such information is usually collected using group-level analysis, which relies on the availability of normative imaging resources (atlases and connectomes). Analysis of DBS data in children with debilitating neurological disorders such as dystonia would benefit from such resources, especially given the developmental differences in neuroimaging data between adults and children. We assembled pediatric normative neuroimaging resources from open-access datasets in order to comply with age-related anatomical and functional differences in pediatric DBS populations. We illustrated their utility in a cohort of children with dystonia treated with pallidal DBS. We aimed to derive a local pallidal sweetspot and explore a connectivity fingerprint associated with pallidal stimulation to exemplify the utility of the assembled imaging resources.

**Methods:**

An average pediatric brain template (the MNI brain template 4.5–18.5 years) was implemented and used to localize the DBS electrodes in 20 patients from the GEPESTIM registry cohort. A pediatric subcortical atlas, analogous to the DISTAL atlas known in DBS research, was also employed to highlight the anatomical structures of interest. A local pallidal sweetspot was modeled, and its degree of overlap with stimulation volumes was calculated as a correlate of individual clinical outcomes. Additionally, a pediatric functional connectome of 100 neurotypical subjects from the Consortium for Reliability and Reproducibility was built to allow network-based analyses and decipher a connectivity fingerprint responsible for the clinical improvements in our cohort.

**Results:**

We successfully implemented a pediatric neuroimaging dataset that will be made available for public use as a tool for DBS analyses. Overlap of stimulation volumes with the identified DBS-sweetspot model correlated significantly with improvement on a local spatial level (R = 0.46, *permuted p* = 0.019). The functional connectivity fingerprint of DBS outcomes was determined to be a network correlate of therapeutic pallidal stimulation in children with dystonia (R = 0.30, *permuted p* = 0.003).

**Conclusions:**

Local sweetspot and distributed network models provide neuroanatomical substrates for DBS-associated clinical outcomes in dystonia using pediatric neuroimaging surrogate data. Implementation of this pediatric neuroimaging dataset might help to improve the practice and pave the road towards a personalized DBS-neuroimaging analyses in pediatric patients.

## Highlights

• Pediatric neuroimaging resources were assembled and implemented to assist deep brain stimulation imaging-based analyses.
• Local sweetspot analysis was performed in a group of pediatric patients with dystonia treated with pallidal DBS.
• Connectomic analysis was performed to demonstrate a distributed network correlate of DBS effect.

## 1 Introduction

Imaging-based analyses of the effects of deep brain stimulation (DBS) have gained popularity over the last decade (Horn and Fox, 2020, Treu et al., 2020). Compared to neuroimaging analyses in adult populations, developmental differences could play a major role in the analysis of pediatric neuroimaging data (Fonov et al., 2011). For example, the basal ganglia and the thalamus widen and elongate with increasing age, in addition to many other morphological changes in gray and white matter (Fonov et al., 2011). There is also an uneven growth of brain structures that cannot be entirely represented using an average adult brain template (Thompson et al., 2000, Gogtay et al., 2004). Thus, mapping the effects of DBS on a group level necessitates use of a common pediatric brain template as a reference (Tödt et al., 2022, Dembek et al., 2019, Horn et al., 2017, Al-Fatly et al., 2019), which has never been adopted in neuroimaging analyses of DBS in children (Coblentz et al., 2021, Tambirajoo et al., 2021, Lumsden et al., 2022). Although transforming a pediatric brain image to an adult brain template is technically possible, the anatomical precision and visualization of basal ganglia structures may differ substantially, especially with regard to localizing DBS electrodes where millimeter differences matter (Horn et al., 2019, Wilke et al., 2002). Furthermore, the use of an adult template could lead to a higher degree of warping and distortion compared to a pediatric template (Wilke et al., 2003, Molfese et al., 2021). Many studies in adult populations have also demonstrated that DBS-associated remote networks can predict clinical improvement (Horn et al., 2017, Al-Fatly et al., 2019, Horn et al., 2022, Ganos et al., 2022, Sobesky et al., 2022). A pediatric connectome that is representative of age-related developmental variances would be beneficial to the application of such predictive network model in children treated with DBS. An example of these variances is increased motor network functional connectivity (particularly in the basal ganglia) during childhood, which correlates with the increasing age of the child (Sussman et al., 2022, Solé-Padullés et al., 2016).

Neurological disorders can affect various age groups. Dystonia is a good example, as it can manifest in different stages throughout life. The disorder is characterized by sustained or intermittent muscle contractions causing abnormal, often repetitive, movements, postures, or both in one or more body regions (Albanese et al., 2013). Clinically, dystonia is defined as isolated dystonia if it is the only feature, or as combined dystonia when associated with other movement disorders. The etiology of dystonia can be inherited, idiopathic, or acquired. Moreover, the pathophysiological background is complex in this network disease, involving abnormal inhibition, plasticity and other features on different levels of the nervous system (Koy et al., 2016, Sanger et al., 2010). Pediatric dystonia is a difficult condition with a negative impact on a patient’s quality of life and on caregivers (van Egmond et al., 2015, Koy et al., 2022). Pharmacotherapy is limited due to the lack of efficacy or intolerable side effects (Lumsden et al., 2016). DBS has been established as a safe and effective treatment alternative for pharmacorefractory dystonia (Volkmann et al., 2012). Multiple studies have investigated the beneficial outcomes of DBS in children with idiopathic or inherited dystonia, with the globus pallidus internus (GPi) as the most common target for electrode implantation (Olaya et al., 2013, Ghosh et al., 2013, Krause et al., 2016). Despite several studies on optimal targeting for the best clinical outcomes in adults, data on how neuroimaging-based analyses could improve targeting of electrodes and clinical outcomes after pallidal DBS in pediatric cohorts are scarce (Coblentz et al., 2021, Tambirajoo et al., 2021, Lumsden et al., 2022). It is therefore of paramount importance to investigate whether such analyses could elucidate the local distribution and remote connections of efficacious pallidal stimulation in children with dystonia.

In our study, we sought to build a set of age-respective, neuroimaging resources for imaging-based analyses in a pediatric DBS population. We used an age-specific, pediatric MNI template to comply with our cohort’s age. The latter allows for accumulating the imaging information of patients into a common stereotactic space and hence eases group-level analyses. We also built a pediatric basal ganglia atlas that suits the pediatric template and better demonstrates basal ganglia nuceli of relevance to pallidal DBS-surgery. Finally, we assembled a pediatric functional connectome from 100 neurotypical children. We wanted to demonstrate the utility of these neuroimaging resources in mapping the clinical effects of pallidal DBS, using DBS-data of 20 children from the German Registry on Pediatric DBS (GEPESTIM) (Koy et al., 2017). The clinical effects of DBS were then locally mapped using a statistical sweetspot model. Electrode localization and sweetspot distribution were visualized in relation to the pediatric basal ganglia atlas. Lastly, a whole-brain connectivity model was estimated by correlating clinical outcomes with stimulation-related connectivity in a voxel-wise fashion using the pediatric functional connectome.

## 2 Methods

### 2.1 Pediatric neuroimaging dataset assembly

To overcome the co-registration/normalization bias that can be introduced by warping pediatric images onto an adult brain template, we incorporated an unbiased pediatric MNI template (Fonov et al., 2011) in Lead-DBS pipelines (Horn et al., 2019). To ensure coverage of the full span of pediatric ages, a template representative of the age group 4.5–18.5 years was chosen. To comply with the routine of spatially normalizing individual brain images into the adult MNI space in Lead-DBS, the asymmetric version of the aforementioned age-range template was chosen. This template has a 1 × 1 × 1 mm resolution and represents an average of 324 enrolled children. In addition, the template is available in multispectral versions (T1, T2 and proton density (PD)). All were included to take advantage of the multispectral option of spatial normalization routines.

Visualizing DBS electrodes in relation to the surrounding anatomical structures is highly important to clinicians and researchers. A pediatric DISTAL atlas was introduced as a new atlas in Lead-DBS, warping specific structures from the adult DISTAL atlas (Ewert et al., 2018) relevant to the current work. First, the pediatric MNI space was co-registered and normalized to the default adult space used in Lead-DBS (MNI152 NLIN 2009b). Manual refinement of the normalization step was performed on structures incorporated in the pediatric DISTAL atlas, using WarpDrive (Oxenford et al., 2022). Specifically, the GPi, globus pallidus externus (GPe), subthalamic nucleus (STN), and red nucleus (RN) were included in the new pediatric atlas as DBS target structures (GPi/GPe) and to further assure quality of alignment (using the STN and RN). In addition to facilitating electrode visualization, this atlas allowed us to precisely assess the relation of locally-mapped DBS effects to anatomical structures of interest (in this case, the GPi/GPe complex) using sweetspot analysis. The resulting inverse warp field was used to extract atlas structures in the pediatric MNI space.

As one of the neuroimaging-based analyses is to delineate the distributed functional network associated with beneficial DBS therapy, we sought to create a normative resting-state functional MRI (rs-fMRI) connectome. To do so, we downloaded and processed an rs-fMRI dataset from the nyu2 sub-cohort of the Consortium for Reliability and Reproducibility (CoRR) (Zuo et al., 2014), which contained neuroimaging data collected from neurotypical adult and pediatric subjects (https://fcon_1000.projects.nitrc.org/indi/CoRR/html/nyu_2.html) (Zuo et al., 2014). Only data from 107 subjects aged 6–18 years were included (an earlier version of the connectome is mentioned in (Neudorfer et al., 2023); for demographics and imaging protocols, please see (Zuo et al., 2014); for scan parameters, see Supplementary Table 1). The participants had been informed by the investigators to rest with their eyes open during the whole scanning period. All participants underwent exhaustive psychometric tests to determine their neurotypical development. MRI scanning was performed during two different sessions on two different dates to conform with the aim of the original study (CoRR). However, for the purpose of our connectome aggregation, we exclusively used data from session 1, as only a few children had completed two sessions.

### 2.2 Normative connectome processing

The anatomical and functional MRI data from each subject were first preprocessed using a collection of tools from different software (namely FSL, SPM, and Lead-Connectome from the Lead-DBS neuroimaging suite, https://www.lead-dbs.org/about/lead-connectome/). Slice time correction (FSL; https://fsl.fmrib.ox.ac.uk) was applied to the data. Realignment and initial motion correction of the rs-fMRI time series was then performed using mcflirt (FSL; https://fsl.fmrib.ox.ac.uk) (Jenkinson et al., 2012). Subjects were excluded if they had a framewise-displacement of >0.5 mm in >50 % of the volumes (Power et al., 2014) (seven subjects were excluded based on this criterion). Detrimental motion effects were regressed out from the data using code implemented in Lead-Connectome (https://www.lead-dbs.org/about/lead-connectome/). Spatial smoothing was performed using a Gaussian kernel of 6 mm full width at half maximum, after which a high-pass filter of 0.01 Hz and a low pass filter of 0.08 Hz were applied to the data to mitigate the effects of scanner drift and high-frequency noise fluctuations,

Finally, we regressed out the average BOLD time series over cerebrospinal fluid (CSF) and white matter (Caballero-Gaudes and Reynolds, 2017). To do this, the corresponding T1-weighted structural image for each subject was segmented using the SPM “*newsegment*” function (Friston et al., 1994). The resultant masks were linearly aligned (coregistered) to the rs-fMRI images, from which masks of white matter, CSF, and gray matter were obtained. The average signal over the CSF and white matter masks was then calculated and regressed from the rs-fMRI time series via linear regression. Regression of the global signal was also performed using Lead-Connectome Matlab code (Fox et al., 2009). Normalization of functional volumes to the pediatric MNI space was then performed using FSL-FNIRT to nonlinearly warp the anatomical T1 images to the pediatric MNI space, and later apply the warp to the coregistered rs-fMRI volumes. Following the normalization of each fMRI acquisition, a 285,903 × 180 matrix – containing the BOLD signal of every voxel (n = 285,903) for each volume in the time series (n = 180) – was computed using Lead-Connectome. The data were then masked to only include voxels within the brain in a readable format for seed-based connectivity analyses in *Lead-Mapper* (another toolbox from Lead-DBS neuroimaging suite).

### 2.3 Study cohort

Twenty children with a diagnosis of dystonia were selected from the GEPESTIM registry. Original trial data was provided by German DBS centers. After screening the available imaging data from the GEPESTIM cohort, the current data for analysis was collected from five different neurological centers across Germany. Reasons for exclusion of data from the original GEPESTIM cohort were lack of pre- and/or postoperative imaging datasets or poor-quality scans (scans with artefacts) that were insufficient for electrode reconstruction and localization, lack of documented DBS settings corresponding to documented clinical scores, and an insufficient period of postoperative clinical follow-up. Ethical approvals were provided by each participating center. Each patient was preoperatively assessed by an expert pediatric neurologist, and at least one follow-up was performed postoperatively at 6 months or later (given a latency of approximately 6 months for pallidal DBS to take effect). A preoperative and postoperative Burke-Fahn-Marsden Dystonia Rating Scale (BFMDRS) score was collected for each patient (Burke et al., 1985), and the improvement under DBS was calculated as the percentage ratio of the difference between these scores. Preoperative MRI scans were also acquired from each respective clinical center, in addition to a postoperative computed tomography (CT) or MRI scan to confirm the final DBS lead locations. There were regular postoperative follow-up visits to try to program the most clinically beneficial DBS settings. We used the most clinically stable DBS programming parameters. Detailed information and trial protocols can be found in the original GEPESTIM registry publication (Koy et al., 2017).

### 2.4 Estimation of electrode localization and stimulation volumes

The DBS electrodes were localized using the open-source software Lead-DBS (https://www.lead-dbs.org) (Horn et al., 2019). The postoperative MRI or CT were co-registered to the corresponding preoperative MRI of each patient using Statistical Parametric Mapping software (SPM12; https://www.fil.ion.ucl.ac.uk/spm/software/spm12/) (Friston et al., 1994) or the Advanced Normalization Tools (ANTs; https://stnava.github.io/ANTs/) (Avants et al., 2008), respectively. The latter tools are integrated within the Lead-DBS software. Next, the co-registered images from all patients were warped to the pediatric MNI space using the ANTs symmetric normalization (SyN) strategy. Both co-registered and normalized images were visualized and quality controlled for any mismatch. Importantly, we used a new feature of Lead-DBS WarpDrive (Neudorfer et al., 2023) to control for any minute differences in specific brain regions that were still misaligned in the pediatric MNI space, by manually warping segments of the image. To minimize effects of brain-shift due to perioperative pneumocephalus, we applied brain-shift correction as implemented within Lead-DBS (Horn et al., 2019). This strategy refines linear mappings between postoperative and preoperative scans using consecutive alignment routines focused on the target regions (basal ganglia). Accordingly, nonlinear shifts introduced by pneumocephalus (usually present in frontal regions, since patients are in a supine position during scans) were substantially minimized. DBS electrodes were automatically pre-reconstructed using the PaCER algorithm (Husch et al., 2018) for postoperative CT and the TRAC/CORE algorithm (Horn et al., 2019) for postoperative MRI, and later manually refined as implemented in Lead-DBS.

**Fig. 1:**
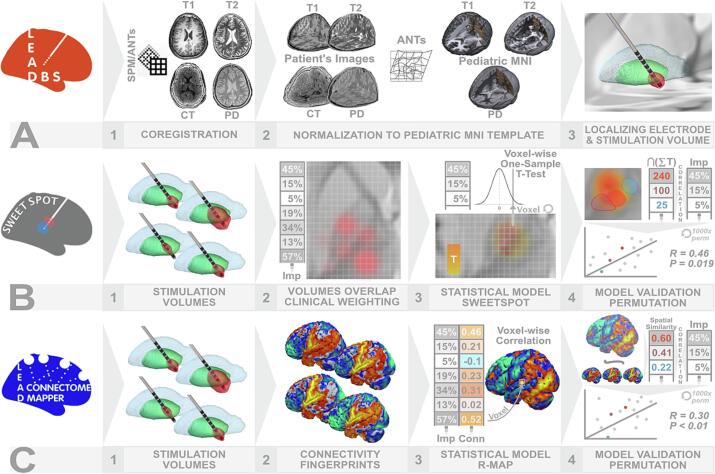
Methodological pipeline summarizing the main steps used in the current study. **A** All available neuroimaging data (pre- and postoperative MRI/CT) of all patients have undergone spatial coregistartion (using SPM/ANTs - A1) in Lead-DBS toolbox. Multispectral normalization was applied using ANTs SyN Diffeomorphic Mapping (A2) implemented in Lead-DBS to ensure a precise warping of all preoperative volumes (T1, T2 and PD when available) to the pediatric MNI template. This spatial normalization was also applied to postoperative CT and followed by 3D reconstruction of DBS electrode model and modeling of stimulation volume (A3). **B** Sweetspot model estimation starts with aggregating stimulation volumes (B1) and weithing them by their respective clinical improvement (B2). Voxel-wise, one-sample *t*-test was then performed to calculate t-score of each voxel stimulated in at least n > 4 patients (20 %) to ensure robust results. This yielded a statistical sweetspot that carries a t-score in each of its voxels (a T-model, B3). Pearson correlation with 1000x permutation was used to validate the sweetspot model (B4) correlating the sum t-scores of the voxels overlapping with each stimulation volume (exemplified by the red, currant and blue volumes of good, medicore and bad responding patients respectively) with their respective percent improvement. **C** Connectivity analysis was carried out using Lead Connectome Mapper. Each stimulation volume (C1) was used as a seed in the 100 subjects, normative pediatric connectome. Connectivity fingerprint maps of all stimulation volumes have been exported (C2) and voxel-wise Pearson correlation between functional connectivity strength and respective percent improvement was calculated (C3) yielding a whole-brain statistical model (R-map). R-map has been validated again by Pearson correlation with 1000x permutation correlating spatial similarities between connectivity fingerprints of stimulation volumes with their respective percent improvement. Conn, connectivity; CT, computed tomography; Imp, improvement; PD, proton density. (For interpretation of the references to colour in this figure legend, the reader is referred to the web version of this article.)

The stimulation volume surrounding active contacts was modeled using the SimBio/FieldTrip approach (Horn et al., 2017) implemented in Lead-DBS. Briefly, the electric fields (E-fields) were estimated in the native space based on the individual optimized stimulation parameters using the finite element method. This was done by solving the static formulation of the Laplace equation on a discretized domain, represented by a tetrahedral four-compartment mesh (composed of gray and white matter, metal and insulating electrode parts). Uniform conductivity of 0.14 S/m was applied to model gray and white matter. We adopted a simplified heuristic strategy, which thresholds electric fields at a vector magnitude above 0.2 V/mm (Åström et al., 2015) and considers the resulting volume as “activated”. Below, we refer to these thresholded volumes as “stimulation volumes”. Volumes were transferred to the pediatric MNI space using the deformation field calculated during spatial normalization. All the steps of the imaging data processing performed in Lead-DBS are illustrated in Fig. 1A.

### 2.5 Sweetspot analysis

A group analysis was performed on stimulation volumes to locally map the effects of DBS in the pallidum. Previous studies used different sweetspot calculation methods (Dembek et al., 2022). In our analysis, we used a recent method that was able to map outcomes in Parkinson’s patients implanted with DBS early in their disease progression (Tödt et al., 2022). Briefly, every binarized mask of each stimulation volume was weighted by its corresponding improvement score. Aggregated together, weighted volumes were statistically tested with a one-sample *t*-test to test the average score of each voxel against zero. The null hypothesis here assumes no effect of DBS on clinical outcomes. This enabled formation of a sweetspot statistical map containing t-scores (T-model). To do so, voxels receiving contribution from less than 20 % of the total stimulation volumes were excluded to ensure robustness of the statistical test. We then calculated the sum of t-scores from the T-model voxels overlapping with each stimulation volume. These sum values were correlated with percentage improvement-scores using a 1000x permutation test (permuting improvement scores). The permutation test was implemented to correct for multiple comparisons and test against null distribution, and is free from assumptions about the distributions, which are typically violated in small sample sizes (Permutation, 2005, Li et al., 2020). We repeated the same analysis on a subgroup of patients that included cases with a diagnosis of inherited or idiopathic dystonia (n = 14). The latter analysis was meant to control for a possible effect of brain lesions in the acquired dystonia cases. For a diagrammatic representation of the sweetspot analysis, please refer to Fig. 1B.

### 2.6 Network analysis

On the whole-brain level, we wanted to test where the DBS electrodes should be connected on a functional network scale to obtain a beneficial DBS outcome. In a similar analysis to previous work (Horn et al., 2017, Al-Fatly et al., 2019), we estimated the functional connectivity profile of each stimulation volume to the rest of the brain using our assembled pediatric normative rs-fMRI connectome. In brief, the average BOLD signal of the bilateral stimulation volumes for each patient was correlated to every other voxel BOLD signal in the brain from each of the connectome subjects. The average voxel-wise R values of 100 subjects were Fisher Z scored to represent the connectivity profile of each patient in our cohort. A voxel-wise correlation of connectivity strengths to the corresponding DBS-related improvement values was then carried out across subjects to yield a statistical R map (Horn et al., 2017). The R map represents the optimal connectivity map, which deciphers important regions of the DBS functional network in children with dystonia. The spatial similarity of each stimulation volume-associated connectivity profile to the R-map was then correlated with a percentage improvement across all 20 subjects using a 1000x permutation test similar to the method described above. Spatial similarities were calculated as the R coefficient of Pearson correlation between the voxel-wise connectivity values in the DBS-connectivity signature map and the voxel-wise R values in the R map. The same analysis was repeated for the subgroup mentioned under the sweetspot analysis section above (inherited/idiopathic subgroup of 14 cases). Fig. 1C summarizes the method used in the network analysis pipeline.

### 2.7 Data availability

Sensitive individual patient data cannot be shared for data protection reasons. All Lead-DBS codes can be accessed on https://github.com/netstim/leaddbs. Neuroimaging resources collected and processed in this manuscript, including the pediatric MNI space/atlas and the connectome, can be downloaded and queried from the Lead-DBS interface.

## 3 Results

### 3.1 Patient characteristics

The patients included in our analysis were part of a cohort from a registered trial (GEPESTIM) (Koy et al., 2017). Five DBS centers from Germany provided data on 20 children who underwent DBS until the age of 18 years between the 2008–2020. In our cohort of 11 males and nine females, the mean age of dystonia onset was 2.9 ± 3.21 years, the mean duration of disease at the time of surgery was 8.65 ± 5.06 years, and the mean age at DBS implantation was 11.55 ± 3.91 years. The average postoperative BFMDRS (56.43 ± 32.95 points) was significantly lower (*t*(19) = -2.99, *p* = 0.007; average percentage improvement 23.89 ± 30.95 %) than the preoperative BFMDRS (71.68 ± 26.51 points). The average time to postoperative follow-up to calculate the percentage improvement was 16.20 ± 12.89 months.

Nine patients were diagnosed with inherited dystonia, six patients suffered from acquired dystonia, and in five patients no underlying cause could be identified (idiopathic dystonia). The etiology of dystonia within the group of inherited dystonia was classified as follows: DYT-TOR1A (n = 3), DYT-KMT2B (n = 1), DYT-SGCE (n = 1), DYT-PRKRA (n = 1), DYT-ANO3 (n = 1), GNAO1 gene mutations (n = 2). Among the group of acquired dystonia, all patients experienced perinatal brain injury, including perinatal asphyxia leading to dystonia. Overall, two patients with acquired and one patient with idiopathic dystonia were preterm (31, 32, and 36 weeks of gestation). In addition, we included one patient with hemi-dystonia, whereas 19 patients had generalized dystonia. Ten patients had isolated dystonia, while the other ten presented with dystonia combined with another movement disorder. Pre- and postoperative BFMDRS scores were available for all 20 patients (see Table 1 for demographics and clinical data; detailed individual patient-related information can be found in Supplementary Table 2; stimulation parameters in Supplementary Table 3).

**Table 1:** Patients demographic and clinical data.

Criteria	N (%) or mean ± SD
Gender (male/female)	11 (55)/9 (45)
Age at onset (years)	2.9 ± 3.21
Age at implantation of DBS-system (years)	11.55 ± 3.91
Duration of disease (years)	8.65 ± 5.06
Etiology of dystonia (inherited/acquired/idiopathic)	9 (45)/6 (30)/5 (25)
Postoperative follow-ups	6.9 ± 2.55
Postoperative surveillance period after initial DBS implantation (years)	5.05 ± 2.7
Baseline BFMDRS score	71.68 ± 26.51
Postoperative BFMDRS score	56.43 ± 32.95
Improvement (%)	23.89 ± 30.59

BFMDRS, Burke-Fahn-Marsden Dystonia Rating Scale; DBS, deep brain stimulation; SD, standard deviation.

### 3.2 Pediatric common brain space and subcortical atlas

**Fig. 2:**
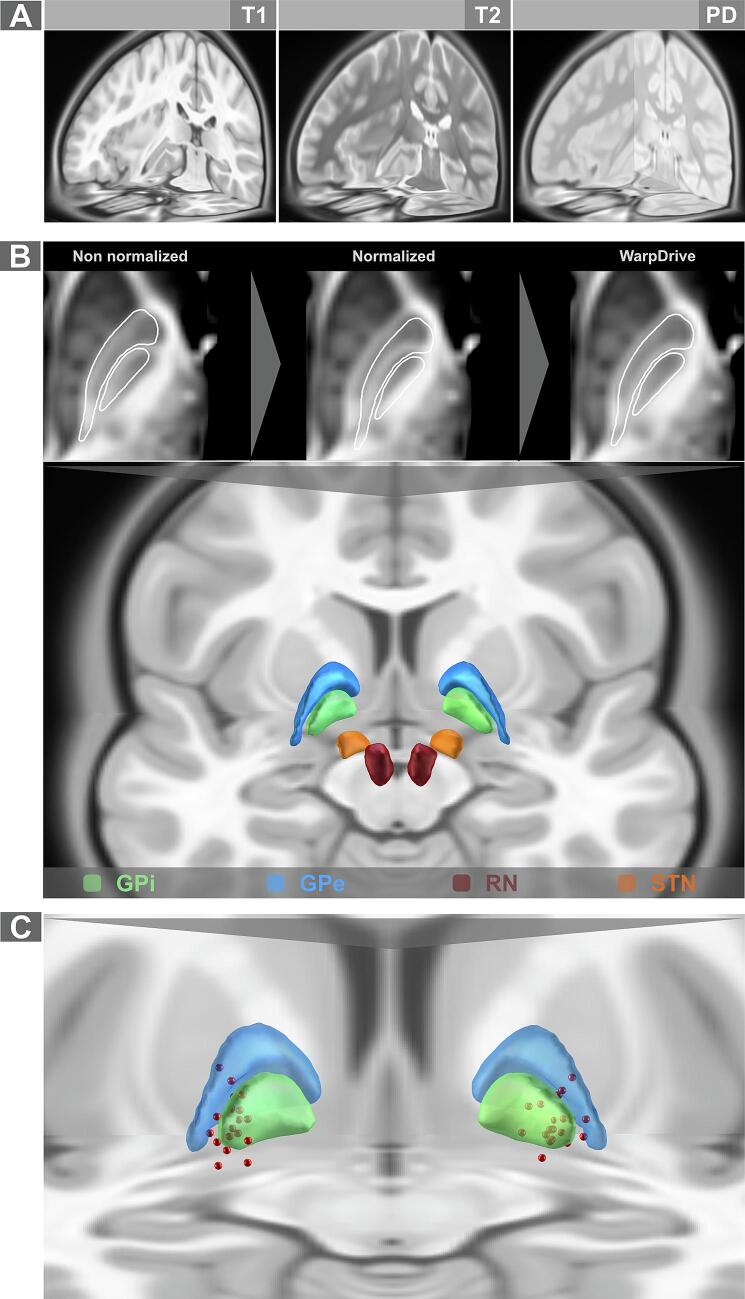
Pediatric standard (MNI) space and subcortical atlas. A. Multispectral MRI acquisitions of the pediatric MNI space (Fonov et al., 2011) were implemented as a standard space in Lead-DBS toolbox. B. Warping of relevant structures from the adult DISTAL atlas (Ewert et al., 2018), with manual fine-tuning using the WarpDrive tool, enabled the pediatric DISTAL atlas to be constructed. C. Active contacts (red spheres) from the GEPSTIM cohort included in our study are depicted in relation to the globus pallidus internus (green) and externus (blue). GPe, globus pallidus externus; GPi, globus pallidus internus; RN, red nucleus; STN, subthalamic nucleus. (For interpretation of the references to colour in this figure legend, the reader is referred to the web version of this article.)

An important step in our study was to implement a common pediatric brain template that could be used in DBS group analysis. A multispectral version of the MNI pediatric space (Fig. 2A) was incorporated and openly distributed within the analysis pipeline of Lead-DBS software, for use in future research. In addition, a pediatric atlas similar to the DISTAL adult atlas was created to highlight important subcortical structures that were surgically targeted or were in the vicinity of the surgical target of DBS for dystonia. Precise transformation of the GPi, GPe, STN, and RN from the DISTAL adult atlas to our pediatric atlas was made possible by using the WarpDrive tool, which enabled manual refinement (Fig. 2B). This in turn ensured precise visualization of the patients’ active contacts in relation to their anatomical structures in space (Fig. 2C).

### 3.3 Degree of overlap with the DBS sweetspot correlates with clinical improvement

**Fig. 3:**
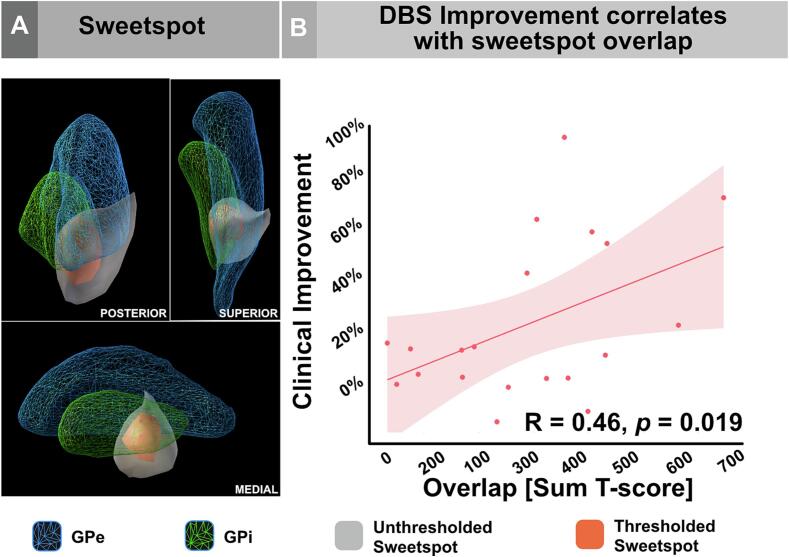
**Sweetspot analysis. A.** Spatial location of the DBS sweetspot in relation to the right GPi/GPe complex from the current pediatric DISTAL atlas. Of note, the sweetspot spanned a region lateral to the GPi on its interface to the GPe in its posterior ventrolateral border. It also extended ventrally to the subpallidal white matter. The sweetspot was presented as a t-score cluster (T-model, see Methods) in unthresholded (gray) and thresholded (red, T ≥ 3) to demonstrate a consistent extent regardless of threshold. **B.** Overlap of the combined DBS stimulation volumes with the sweetspot (T-model) on local level correlated significantly with the DBS-ON clinical improvement. The latter correlation was validated by permuting the percentage improvement scores 1000 times. DBS, deep brain stimulation; GPe, globus pallidus externus; GPi, globus pallidus internus. (For interpretation of the references to colour in this figure legend, the reader is referred to the web version of this article.)

The calculated statistical model (T-model) of the sweetspot was found to lie in the region of the GPi, with a tendency to encroach on the ventral and lateral border of the GPi (on its interface with the GPe) and slightly extending toward the subpallidal region (see Fig. 3A). Overlap of each combined bilateral stimulation volumes with the sweetspot correlated with the corresponding DBS-associated clinical improvement (R = 0.46, *permuted p* = 0.019, Fig. 3B). The results were stable when the analysis was repeated in inherited/idiopathic dystonia subgroup (R = 0.79, *permuted p* = 0.001; Supplementary Fig. 1). The center of gravity coordinates of the sweetspots of the whole- and the idiopathic/inherited-group are x = 21.6, y = −7.44, z = −4.99 and × = 20.2, y = −6.84, z = −5.63, respectively. It should be noted that these coordinates are based on the pediatric MNI template used in the current work and could not be directly compared to an adult template-based results.

### 3.4 A pediatric functional connectome

**Fig. 4:**
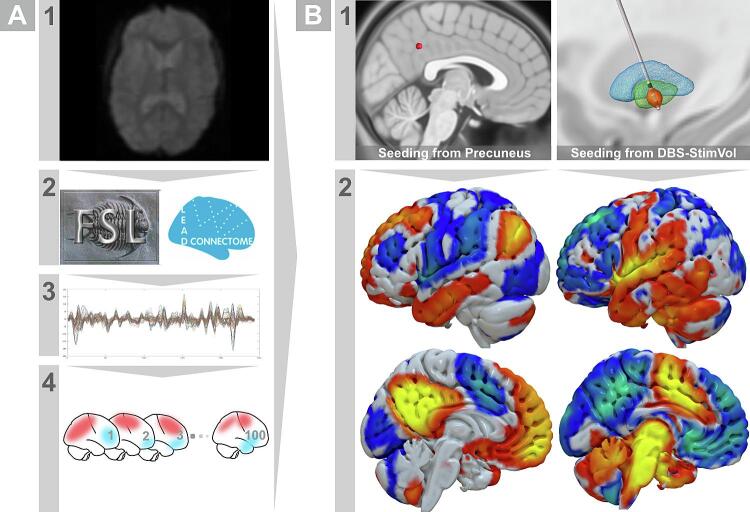
**Resting-state normative pediatric connectome. A.** Pipeline for connectome pre-processing. Raw fMRI scans (1) were downloaded and preprocessed with associated T1 MRI using FSL and Lead-Connectome tools (2) to extract BOLD signals (3) across 100 neurotypical children. Matrices of time-series were stored in a form usable by Lead-Mapper tool for seed connectivity analysis in each subject of the connectome (4). **B.** Estimation of average seed connectivity. (1) Example seed locations from the precuneus and a stimulation volume modeled from one of the patients in our study cohort. (2) Using the Lead-Mapper tool, estimation of seed connectivity profiles (averaged across 100 subjects in the connectome) successfully replicated the default mode network in case of the precuneal seed, and relevant connectivity from GPi region was calculated using stimulation volume as a seed. DBS, deep brain stimulation; GPi, globus pallidus internus.

rs-fMRI data from normally developing children were successfully processed and assembled as a ready-to-use functional connectome for seed-based connectivity (Fig. 4). To pretest the robustness of the connectome, we seeded from a precuneal location (spherical seed size, 4 mm). The seed was manually placed using Mango software (https://www.nitrc.org/projects/mango/) and later fed to Lead-Mapper to estimate the related-connectivity profile. The latter was in agreement with the canonical pattern of the default mode network (Fox et al., 2005). This was a confirmatory step to ensure the vigor of the connectome. In addition, stimulation-related connectivity profiles were visually inspected to give an example of pallidal functional connectivity (see Fig. 4B). It is worth noting that the time-series data of this rs-fMRI connectome were sampled in the pediatric MNI space.

### 3.5 Connectivity pattern of DBS effect

**Fig. 5:**
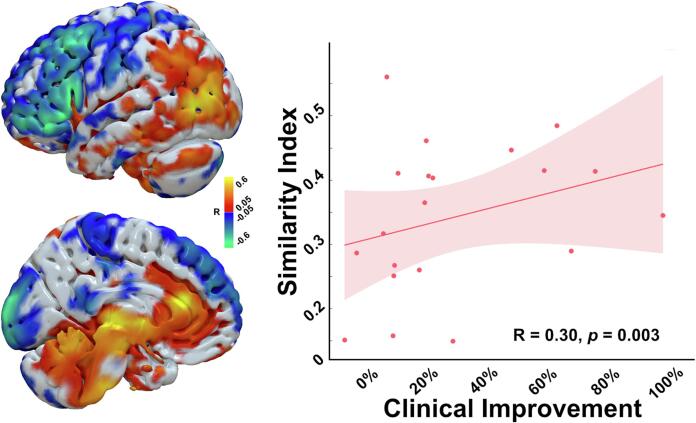
**Functional connectivity fingerprint of beneficial pallidal DBS.** R-map DBS-network model projected on a surface mesh of pediatric MNI space showing different regions and their relevance to DBS outcome. Similarity of the DBS site-associated whole-brain connectivity correlated significantly with clinical improvement across patients in our cohort. DBS, deep brain stimulation.

To obtain further insight from a network perspective, a connectivity analysis was performed harnessing data from the pediatric functional connectome. While the sweetspot analysis looked at the clinical effects of DBS from an electrode localization perspective, functional network analysis extends this concept and extracts related information from whole-brain regions that are indirectly and remotely (polysynaptically) connected to the DBS electrode location. In this regard, the estimated group-level R map demonstrated a peculiar topology where connectivity to areas like the sensorimotor cortex, frontal cortex, and posterior cerebellum was negatively correlated with the clinical effects of DBS (i.e., patients would worsen if their DBS electrodes were more connected to these regions). On the other hand, connectivity to the parietal and anterior cingulate cortices were positively correlated with postoperative improvement. The brainstem and medial and superior parts of the cerebellum also displayed a similar pattern of connectivity that correlated with improvement. In general, the similarity of the individual stimulation-related connectivity profile to the R-map model was significantly positively correlated with DBS improvement across our study cohort (R = 0.30, *permuted p* = 0.003; Fig. 5). The model also yielded significant correlation using permutation testing when repeated on the inherited/idiopathic subgroup (R = 0.40, *permuted p* = 0.001; Supplementary Fig. 2).

## 4 Discussion

Our study aimed to implement a pediatric neuroimaging dataset and showcase the local and remote network correlates of the therapeutic effects of DBS in children with dystonia included in a previous clinical trial (GEPESTIM) (Koy et al., 2017). This allowed us to describe a sweetspot statistical model representative of the clinical effects of DBS by inferring a relationship between stimulation location and clinical improvement in these patients. In addition, a functional-network correlate of effective DBS therapy was elucidated. Taken together, the current findings shed light into how local and remote information can be exploited to explore DBS outcomes in children using pediatric neuroimaging analysis tools.

### 4.1 Towards a refined neuroimaging approach in pediatric DBS

The current understanding of neurological diseases and their therapies is highly dependent on group analysis of data. When neuroimaging data are considered, a common brain template is normally used to aggregate data and perform statistical inference (Treu et al., 2020). To this end, the use of an age-specific brain template will allow better estimation of developmental differences across age groups and account for any anatomical variance (Treu et al., 2020, Gogtay et al., 2004, Lenroot and Giedd, 2006). The use of the pediatric MNI template in our study is a good example. To the best of our knowledge, our study is the first to analyze DBS-related pediatric neuroimaging data using specifically pediatric imaging resources. The use of such a template also helps to minimize the amount of spatial deformation needed to warp the pediatric images to adult templates (Wilke et al., 2003, Molfese et al., 2021, Wilke et al., 2002). Additionally, we minimized the need for manual WarpDrive refinement and maximized structural alignment by using the pediatric MNI template when compared to the adult one as depicted in Supplementary Fig. 3. Although the WarpDrive tool has helped in mitigating minor local misalignments in the normalization, severe atrophied/degenerated structures could still be difficult to refine. However, we did not have such cases in our cohort.

The implementation of the pediatric MNI template has also enabled an atlas of subcortical structures (namely the DISTAL atlas) (Ewert et al., 2018) from the adult MNI space to be adjusted to the pediatric atlas. This atlas contained the main DBS target commonly used to treat childhood dystonia, namely the GPi/GPe complex. These and further subcortical structures (STN and RN) worked as anchor points to refine spatial normalization using the Lead-DBS toolbox (Horn et al., 2019).

Given the current shift from location- to network-based understanding of neurological diseases and treatment options (Fox, 2018), the application of a normative pediatric functional connectome helps to advance an age-relevant connectomic approach in pediatric DBS studies. As structural and functional changes occur during development, connectivity patterns also change and reshape until the brain reaches maturity (Sussman et al., 2022, Solé-Padullés et al., 2016). While previous work relied on adult connectomes analyses (Coblentz et al., 2021, Tambirajoo et al., 2021), our pediatric connectome could facilitate future use of such a connectome in DBS and other pediatric research applications (for instance, mapping networks associated with specific lesions causing childhood neurological diseases) (Cohen et al., 2021). Furthermore, whole-brain mapping of the therapeutic effects of DBS allows a common network to be estimated that can also be targeted by other invasive and non-invasive neuromodulatory techniques (Fox et al., 2014).

Although we used a set of neuroimaging resources that covers the full age-range of our cohort (age at DBS implant – see Supplementary Table 2), future studies could benefit from an age-binned template/connectome to precisely suits the age-range of specific groups of patients. Furthermore, muti-templates (multi-atlases) approaches could also be useful in such scenarios although technically challenging due to difficulties in inter-templates images fusion (Phan et al., 2018). Taken together, the use of all three neuroimaging resources in our work (a template, an atlas, and a connectome) is a step closer toward personalized neuroimaging analysis in pediatric DBS research (Rajamani et al., 2022).

### 4.2 A pallidal DBS sweetspot in children

Antidystonic pallidal sweetspots have previously been described in many adult DBS studies (Horn et al., 2022, Reich et al., 2019, Neumann et al., 2017, Schönecker et al., 2015, Okromelidze et al., 2020). Two recent studies reported an efficacious stimulation site in dystonic children implanted in the GPi using different methodologies (Coblentz et al., 2021, Lumsden et al., 2022). Of note, our sweetspot methodology has been recently used in an adult STN-DBS study (Tödt et al., 2022). Crucially, overlap of the stimulation volume with the sweetspot “T-model” was significantly correlated with DBS-associated improvement across our cohort. This finding emphasizes the importance of treating the output of sweetspot methodology as a statistical model rather than a mere binary location (Reich et al., 2019). The anatomical distribution of the sweetspot in the posteroventral GPi indicated a lateral location in the interface between GPi and GPe. This could explain the importance of the role of the GPe and pallidal input fibers to the GPi in antidystonic DBS effects (Raghu et al., 2021). However, it is difficult to hypothesize the implication of fiber tracts, since our study did not use tractography as a method. Further experiments on how the effects of DBS can be relayed through fibers in the vicinity of the pallidum in children are needed, given the possible developmental differences compared to adults (Feldman et al., 2010). Nevertheless, the proper use of a normative structural connectome or atlas should be valuable, in addition to the well-acquired, native diffusion MRI of the patients. Another aspect is the ventral extension of the sweetspot to the subpallidal white matter. The latter could, to some degree, be regarded in line with the findings from a big adult cohort (Reich et al., 2019), especially when compared to the results of generalized dystonia in the study by Horn et al (Horn et al., 2022). Of note, previously published sweetspots were calculated using different statistical tests and were derived from different types of dystonia in adult populations. An example is the above-mentioned study by Horn et al where a voxel-wise correlation between e-field magnitude and percent improvement was used to estimate a sweetspot model.

### 4.3 A functional connectivity correlate of antidystonic DBS effects in pediatric patients

As already mentioned, the use of network mapping of symptoms induced by brain lesions (Fox, 2018) – or in our case, mapping dystonia improvements induced by DBS – has gained increasing interest in recent years (Horn et al., 2022, Horn and Fox, 2020). Apart from understanding the link between network modulation and disease pathophysiology on a distributed brain topology, network mapping allows efficient translation between different invasive and non-invasive therapeutic strategies by targeting common connectivity substrates (Fox et al., 2014). Currently, there is limited applicability of non-invasive neuromodulation in children, and the concept of a common network target could be used in future trials in children. Our study highlighted a functional connectomic fingerprint that is partially in line with a relevant topology of previous work (Horn et al., 2022). Specifically, the negatively-correlated sensorimotor cortices were a central finding in multiple studies (Horn et al., 2022). Furthermore, the somatosensory cortex has repetitively been shown to have a major influence on the mechanism of dystonia, regardless of somatotopic distribution (focal or generalized) (Kojovic et al., 2013). Studies have demonstrated the role of cerebellar neuromodulation when mitigating dystonic symptoms with different therapies (Koch et al., 2014, Pizoli et al., 2002, Prudente et al., 2013). While the anterior cingulate cortex is not an obvious location associated with movement disorders, its contribution to motor control has been proved in primates and human behaviour (Paus, 2001). The connectomic fingerprint illustrated in our study overlaps partially with a recent one by Horn et al (Horn et al., 2022), which used an adult functional connectome to map the therapeutic effects of DBS in an adult dystonic cohort. However, some differences in the topology could be due to the inherent characteristics of the study cohort (heterogeneous types of dystonia). Another explanation could be that the DBS-related connectivity pattern is age dependent. This, however, should remain highly speculative until a proper systematic analysis between adult and pediatric DBS cohorts using respective imaging resources has been performed.

### 4.4 Limitations

The small sample size of the dystonia cohort included in our study is a limitation. However, dystonia is classified as a rare disease and its occurrence in childhood adds to the difficulties (Fernández-Alvarez and Nardocci, 2012). This aspect can be mitigated through collaboration with different clinical centers to overcome the problem of small cohorts. We strived to use all possible data from our cohort to maximize the number of participants included. Secondly, our cohort consisted of patients with different types of dystonia and different ages at surgery. These factors might have added to the heterogeneity of the data, especially when considering that the effects of DBS depend on the underlying pathophysiology (Kupsch et al., 2003, Ellis, 2011). We accounted for the possible influence of acquired dystonia by repeating the sweetspot and network analyses after excluding them. Further bigger cohorts should test the latter assumption using a more systematic approach. We did not analyze data from cases with acquired dystonia as a separate group in our sweetspot and network models due to lack of sufficient number of patients (N = 6). A forthcoming task would be to perform such analyses in larger groups of patients taken into consideration the heterogeneity of the causative factors. However, heterogeneity in the etiologies and body distribution of dystonic manifestations should always be taken into account especially in relation to functional therapeutic networks. An example is the study of Horn et al (Horn et al., 2022), where different networks were identified depending on the bases of the distribution of dystonia (cervical vs generalized).

Thirdly, the BFMDRS score was initially developed for adults with isolated dystonia (Burke et al., 1985). As patients with acquired dystonia often have a complex hyperkinetic movement disorder, encompassing choreoathetosis and ballism, the BFMDRS does not capture all aspects of the clinical picture. Even in patients with little or even absent observable changes in the BFMDRS, DBS can improve domains such as function and quality of life. Therefore, the sole use of the BFMDRS is insufficient to fully assess DBS effects in these patients (Gimeno et al., 2012). Accordingly, the use of multidisciplinary assessments of motor and non-motor domains should be aimed for in future studies in order to catch the full DBS effects in patients with acquired dystonia. Nevertheless, our data should be seen as a foundation to the technical approach in pediatric DBS patients, although associated results should also be interpreted with caution. A fourth limitation is the implementation of a normative connectome for DBS network mapping. Although the use of such connectomes has shown good performance in explaining the network effects of DBS in different studies (Horn et al., 2017, Al-Fatly et al., 2019, Horn et al., 2022, Ganos et al., 2022, Sobesky et al., 2022), a possible comparison to patient-specific connectivity data is of importance (Wang et al., 2021) as some patients have brain lesions that could impact the network. However, publicly-available data are usually collected for research purposes and are of higher resolution than clinically-collected data. Another specific limitation in children with movement disorders like dystonia is movement artefacts (Sussman et al., 2022). The latter could also favor the use of a more stable normative connectome. Additionally, average seed-based connectivity profiles derived from multiple subjects in a normative connectome can enhance the signal-to-noise ratio. Finally, the occurrence of other clinical features like epilepsy in GNAO1 cases (N = 2) could play additional roles defining the therapeutic networks. However, we focused on the improvement in BFMDRS as a clinical measurement for the severity of dystonia in our patients and used it as an independent variable to exclusively determine the “anti-dystonic” sweetspot and network. However, we focused on the percentage improvement in BFMDRS as a clinical measurement for the severity of dystonia in our patients and used it as an independent variable to exclusively determine the “anti-dystonic” sweetspot and network.

## 5 Conclusions

We used a set of pediatric resources to perform neuroimaging analyses on a group level in pediatric DBS patients. This enabled us to identify a sweetspot of beneficial pallidal DBS effects using a pediatric template and atlas, as well as an optimal whole-brain functional connectomic network using a pediatric normative connectome. The latter corresponds to current knowledge about the pathophysiologic network model responsible for dystonia. Our findings confirm previous results and will facilitate future neuroimaging analyses in pediatric DBS research.

## Funding

AK receives a grant by Dr. Rita and Dr. Hans Günther Herfort Stiftung, the institution of AK receives a grant by Boston Scientific. AAK received travel grants and honoraria from Medtronic and Boston Scientific. LT received occasional payments as a consultant for Boston Scientific between September 2021-September 2022. LT received honoraria as a speaker on symposia sponsored by Boston Scientific, AbbVIE, Novartis, Neuraxpharm, Teva, the Movement Disorders Society and DIAPLAN. VVV received payments from Boston Scientific for lectures and advisory boards. TAD was supported by the Cologne Clinician Scientist Program (CCSP)/Faculty of Medicine/University of Cologne, funded by the German Research Foundation (DFG, FI 773/15-1).

## CRediT authorship contribution statement

**Bassam Al-Fatly:** Conceptualization, Data curation, Formal analysis, Methodology, Software, Visualization, Writing – original draft. **Sabina J. Giesler:** Data curation, Investigation, Resources, Writing – review & editing. **Simon Oxenford:** Methodology, Software, Writing – review & editing. **Ningfei Li:** Methodology, Software, Writing – review & editing. **Till A. Dembek:** Project administration, Resources, Writing – review & editing. **Johannes Achtzehn:** Formal analysis, Resources, Writing – review & editing. **Patricia Krause:** Data curation, Investigation, Writing – review & editing. **Veerle Visser-Vandewalle:** Data curation, Investigation, Writing – review & editing. **Joachim K. Krauss:** Data curation, Investigation, Writing – review & editing. **Joachim Runge:** Data curation, Investigation, Writing – review & editing. **Vera Tadic:** Data curation, Investigation, Writing – review & editing. **Tobias Bäumer:** Data curation, Investigation, Writing – review & editing. **Alfons Schnitzler:** Data curation, Investigation, Writing – review & editing. **Jan Vesper:** Data curation, Investigation, Writing – review & editing. **Jochen Wirths:** Data curation, Investigation, Writing – review & editing. **Lars Timmermann:** Project administration, Writing – review & editing. **Andrea A. Kühn:** Conceptualization, Funding acquisition, Project administration, Supervision, Writing – review & editing. **Anne Koy:** Conceptualization, Funding acquisition, Project administration, Supervision, Writing – review & editing.

## Declaration of Competing Interest

AAK received honoraria from Medtronic and Boston Scientific, not related to this work. AK is a principal investigator in the STIM-CP trial, partly sponsored by Boston Scientific. JKK is a consultant to Medtronic, Boston Scientific, aleva and Inomed. LT serves as the vice president of the German Neurological Society. TAD has received speaker honoraria from Medtronic & Boston Scientific.

The remaining authors declare that they have no known competing financial interests or personal relationships that could have appeared to influence the work reported in this paper.

## Data Availability

Sensitive individual patients’ data cannot be shared for data protection reasons. Neuroimaging resources collected and processed in this manuscript can be downloaded and queried from Lead-DBS GUI.
