# Supplementary material for: Neuroimaging-based analysis of DBS outcomes in pediatric dystonia: Insights from the GEPESTIM registry
Source: Neuroimage Clin. 2023 Jun 10;39:103449. doi: 10.1016/j.nicl.2023.103449 (PMC10275720; doi:10.1016/j.nicl.2023.103449)
Supplement: Supplementary data 1 [file mmc1.docx]

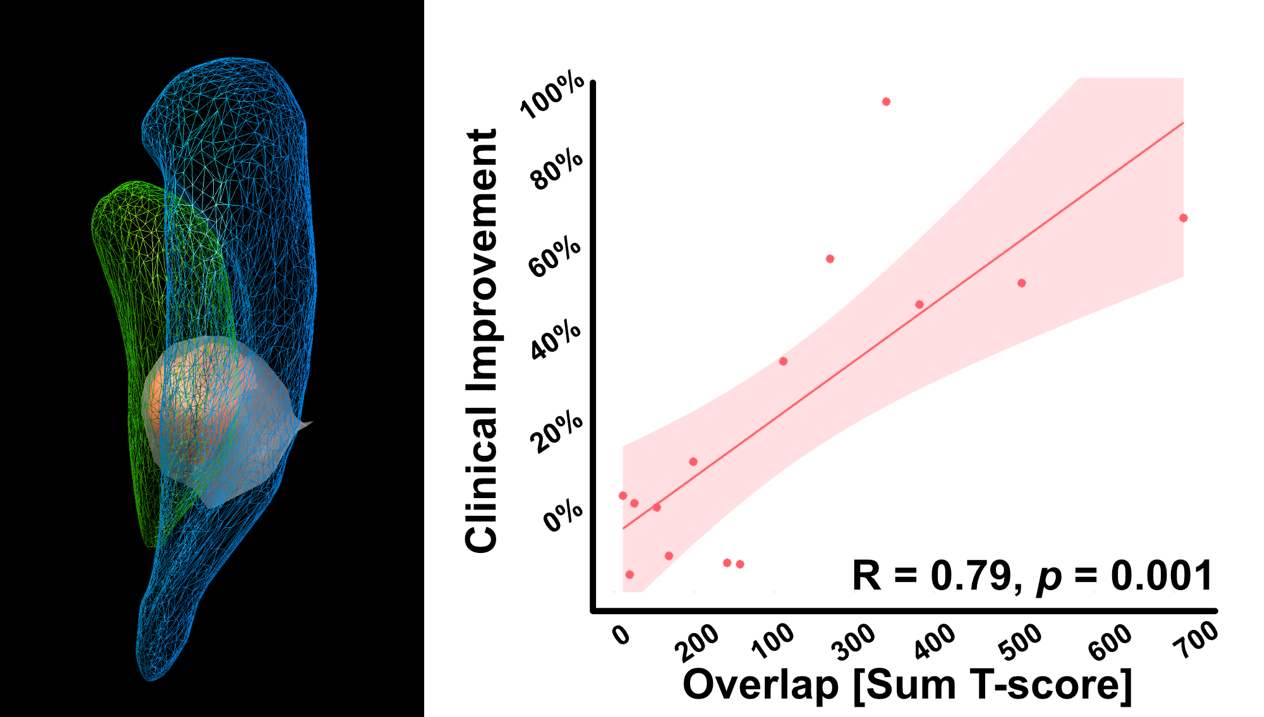


**Supplementary Figure 1**

Sweetspot overlap correlation for inherited and idiopathic cases. Left panel shows unthresholded sweetspot T-model in grey and thresholded (t > 2) in red together with a wireframe GPi (green) and GPe (blue) in pediatric MNI space.


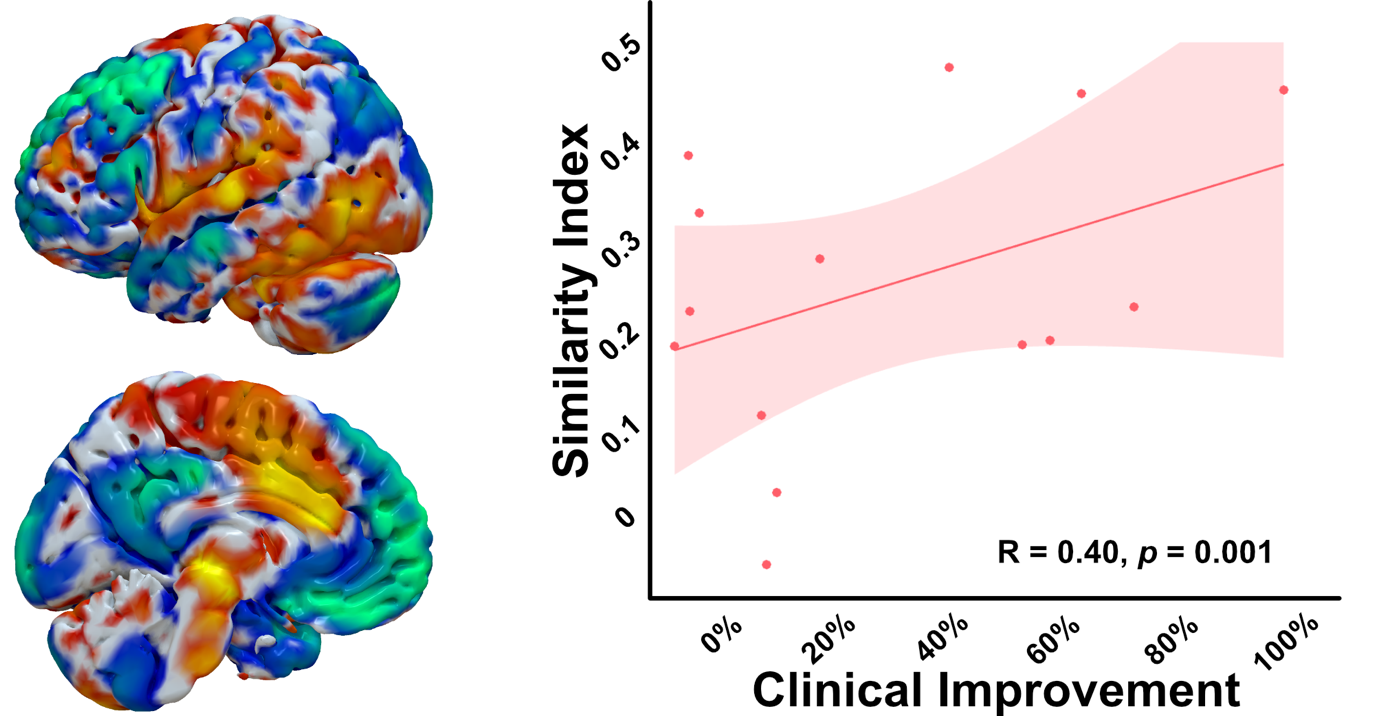


**Supplementary Figure 2**

Network correlation for inherited and idiopathic dystonia cases. Left panel shows the functional network correlate overlaid on a pediatric template surface model.


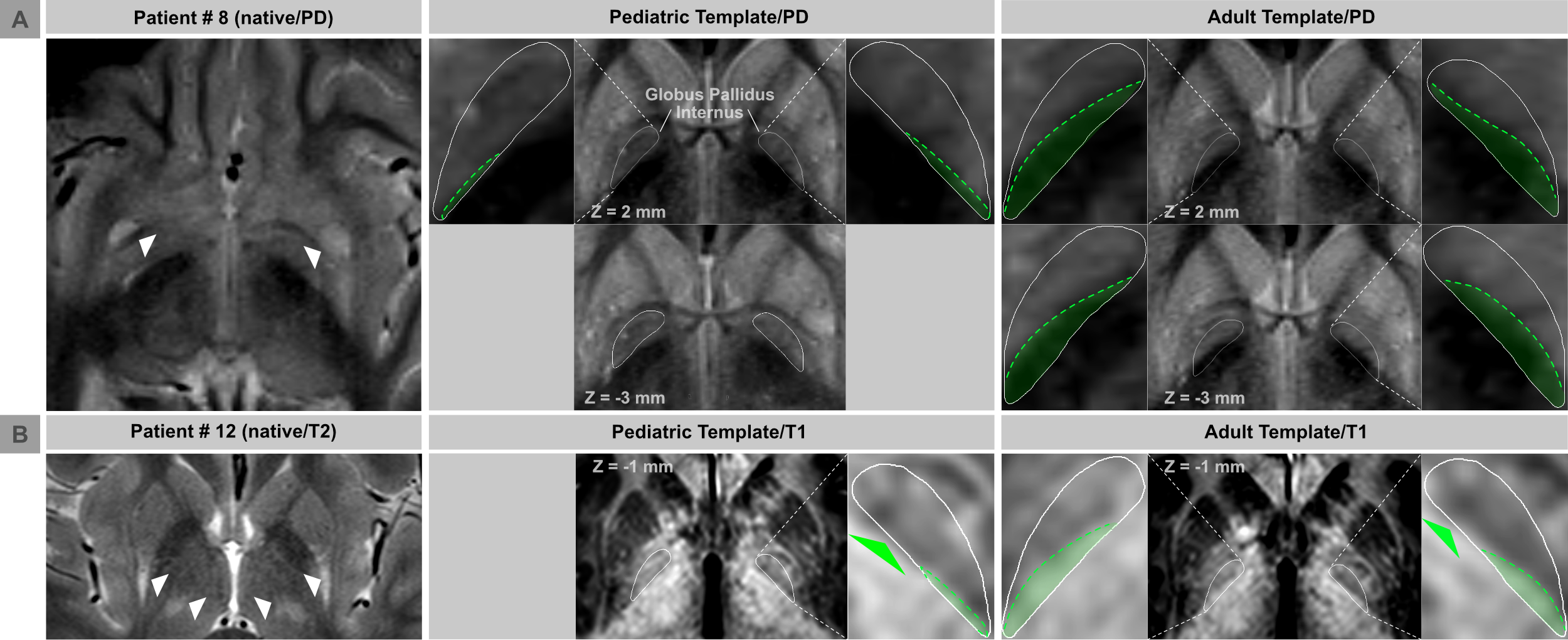


**Supplementary Figure 3**

Differences in normalization performance and structural alignment using pediatric and adult MNI templates. ANTs normalization algorithm was used and the contour of the GPi is shown in white as a DBS target of interest from the corresponding-template, DISTAL atlases. The degree of atlas-defined GPi misalignment with the underlying image (PD images for patient # 8 in A and T1 images for patient # 12 in B) can already be visually evaluated from the green-shaded areas. Obviously, the use of the pediatric template yielded better alignment results when compared to that of the adult counterpart. Even in patient # 12 (B), where anterior GPI misalignment were similar in both templates (green arrows), the degree of posterior GPi misalignment, where the electrode is usually implanted, was less in the pediatric template. Both patients had lesions (white arrows) in the region of the striatum (A, PD image in native space) and posterior putamen and thalamus (B, T2 image in native space) as shown in left-most panels. These lesions could also put more challenges to correctly warp affected regions to the respective template. This has been well mitigated when using the pediatric template as compared to the adult one.

ANTs, Advanced Normalization Tools; GPi, globus pallidus internus; PD, proton density.
