# Supplementary material for: Neuroimaging-based analysis of DBS outcomes in pediatric dystonia: Insights from the GEPESTIM registry
Source: Neuroimage Clin. 2023 Jun 10;39:103449. doi: 10.1016/j.nicl.2023.103449 (PMC10275720; doi:10.1016/j.nicl.2023.103449)
Supplement: Supplementary data 3 [file mmc3.docx]

**Supplementary Table 1**

MRI scanning parameters of the participants in the pediatric connectome.

| **T1 MRI** | | | | | | | | | | | | | | | | | | | | | |
| --- | --- | --- | --- | --- | --- | --- | --- | --- | --- | --- | --- | --- | --- | --- | --- | --- | --- | --- | --- | --- | --- |
| **Manufacturer** | **Headcoil** | **Field Strength** | **Sequence** | **Flip Angle [Deg]** | **Inversion Time (TI) [ms]** | **Echo Time (TE) [ms]** | **Repetition Time (TR) [ms]** | **Bandwidth per Voxel (Readout) [Hz]** | **Parallel Acquisition** | **Number of Slices** | | **Slice Thickness [mm]** | | **Slice Gap [mm]** | | **Field of View [mm]** | **Acquisition Matrix** | | **Slice In-Place Resolution [mm2]** | **Acquisition Time [min:sec]** | |
| Siemens | Allegra | 3T | 3D MPRAGE | 7 | 1,100 | 3.25 | 2,530 | 200 | Off | 128 | | 1.3 | | 0.65 | | 256 | 256×192 | | 1.3×1.0 | 08:07 | |
| **rsfMRI** | | | | | | | | | | | | | | | | | | | | | |
| **Manufacturer** | **Model** | **Headcoil** | **Field Strength** | **Sequence** | **Flip Angle [Deg]** | **Echo Time (TE) [ms]** | **Repetition Time (TR) [ms]** | **Bandwidth per Voxel (Readout) [Hz]** | **Number of Slices** | **Slice Thickness [mm]** | **Slice Gap [mm]** | | **Field of View [mm]** | | **Acquisition Matrix** | | **Slice In-Place Resolution [mm2]** | **Number of Measurements** | | | **Acquisition Time [min:sec]** |
| Siemens | Allegra | 1 Chan | 3T | EPI | 90 | 15 | 2,000 | 3,906 | 33 | 4 | 0 | | 240 | | 80×80 | | 3.0×3.0 | 180 | | | 06:00 |

**Supplementary Table 2**

Patients’ characteristics.

| **Patient** | **Gender** | **Etiology** | **When Inherited:  Genetics** | **Manifestation** | **Anatomic distribution** | **Phenom-enology** | **Age at dystonia onset (years)** | **Age at DBS-implant (years)** | **Disease duration at DBS-implant (years)** | **Pre-/Post-operative**  **BFMDRS** | **% Impro-vement** |
| --- | --- | --- | --- | --- | --- | --- | --- | --- | --- | --- | --- |
|  |  |  | **When Acquired:  Further information** |  |  |  |  |  |  |  |  |
| 1 | ♀ | Acquired | Perinatal brain injury (perinatal asphyxia) | C (choreoatheto-ses) | G | P-T | 2 | 10 | 8 | 89/86 | 3.37 |
|  |  |  |  |  | Head, mouth, trunk, left arm, right arm, left leg, right leg |  |  |  |  |  |  |
| 2 | ♂ | Inherited | DYT-SGCE | C  (myoclonus) | G | P-T | 3 | 17 | 14 | 26/10.5 | 59.62 |
|  |  |  |  |  | Neck, trunk, left arm, right arm |  |  |  |  |  |  |
| 3 | ♂ | Inherited | DYT-TOR1A | NA | F | T | 11 | 13 | 2 | 28/24 | 14.29 |
|  |  |  |  |  | Trunk, Left leg, Right leg |  |  |  |  |  |  |
| 4 | ♂ | Idiopathic |  | I | G | P-T | 6 | 14 | 8 | 24/13.5 | 43.75 |
|  |  |  |  |  | Neck, head, mouth, trunk, left arm, right arm, left leg, right leg |  |  |  |  |  |  |
| 5 | ♂ | Inherited | ~~DYT-PRKRA~~ | I | G | T | 4 | 13 | 9 | 92/25 | 72.83 |
|  |  |  |  |  | Dystonic storm ~~Trunk, right arm, right leg~~ |  |  |  |  |  |  |
| 6 | ♀ | Inherited | DYT-TOR1A | I | G | P-T | 5 | 7 | 2 | 62/22 | 64.52 |
|  |  |  |  |  | Head, left arm, right arm, left leg, right leg |  |  |  |  |  |  |
| 7 | ♀ | Inherited | DYT-KMT2B | C  (tremor, myoclonus,) | G | P-T | 4 | 9 | 5 | 96/43 | 55.21 |
|  |  |  |  |  | Left arm, right arm, right leg |  |  |  |  |  |  |
| 8 | ♀ | Acquired | Perinatal brain injury | C (+choreoathet-osis) | G | P-T | 0 | 16 | 16 | 82.5/94 | -13.94 |
|  |  |  |  |  | Trunk, right arm, left arm, right leg, left leg |  |  |  |  |  |  |
| 9 | ♀ | Idiopathic |  | I | H | T | 7 | 12 | 5 | 34/26 | 23.53 |
|  |  |  |  |  | Left arm, left leg |  |  |  |  |  |  |
| 10 | ♂ | Idiopathic |  | I | G | T | 1 | 11 | 10 | 97/94 | 3.09 |
|  |  |  |  |  | Head, eyes, mouth, neck, trunk, left arm, right arm, left leg, right leg |  |  |  |  |  |  |
| 11 | ♂ | Acquired | Perinatal brain injury, infarction of the A. cerebri media left | C (+choreoathet-oses, dysarthria) | G | P-T | 0 | 14 | 14 | 50.5/44.5 | 11.88 |
|  |  |  |  |  | Head, eyes, mouth, neck, trunk, left arm, right arm, left leg, right leg |  |  |  |  |  |  |
| 12 | ♂ | Acquired | Perinatal brain injury | C (+choreoathet-oses,) | G | P-T | 0 | 14 | 14 | 71.5/78.5 | -9.79 |
|  |  |  |  |  | Head, eyes, mouth, neck, trunk, left arm, right arm, left leg, right leg |  |  |  |  |  |  |
| 13 | ♂ | Inherited | DYT-TOR1A | I | G | P-T | 6 | 7 | 1 | 68/2.5 | 96.32 |
|  |  |  |  |  | Left arm, right leg |  |  |  |  |  |  |
| 14 | ♂ | Acquired | Cerebellar parenchymal defect of undetermined genesis (detected on preoperative cerebral imaging) | I | G | T | 1 | 16 | 15 | 112/96.5 | 13.84 |
|  |  |  |  |  | Head, eyes, mouth, neck, trunk, left arm, right arm, left leg, right leg |  |  |  |  |  |  |
| 15 | ♀ | Inherited | DYT-GNAO1 | Combined (+ choreoathetoses, central hypotonia, global developmental delay, ~~epilepsy~~ **status post neonatal epileptic seizures**) | G | P-T | 0 | 14 | 14 | 87.5/85 | 2.86 |
|  |  |  |  |  | Trunk, left arm, left leg, right arm, right leg |  |  |  |  |  |  |
| 16 | ♂ | Idiopathic |  | I | G | T | 7 | 13 | 6 | 66/56 | 15.15 |
|  |  |  |  |  | Head, neck, trunk, left arm, right arm, left leg, right leg |  |  |  |  |  |  |
| 17 | ♀ | Idiopathic |  | I | G | T | 0 | 15 | 15 | 96/80 | 16.67 |
|  |  |  |  |  | head, neck, trunk, left arm, right arm, left leg, right leg |  |  |  |  |  |  |
| 18 | ♀ | Acquired | Perinatal brain injury | C (+choreoathetoses, central hypotonia) | G | P-T | 0 | 8 | 8 | 87.5/88 | -0.57 |
|  |  |  |  |  | Head, neck, trunk, left arm, right arm, left leg |  |  |  |  |  |  |
| 19 | ♂ | Inherited | DYT-ANO3 | I | G | P-T | 1 | 5 | 4 | 88.5/84.5 | 4.52 |
|  |  |  |  |  | Head, eyes, mouth, trunk, left leg, right leg |  |  |  |  |  |  |
| 20 | ♀ | Inherited | DYT-GNAO1 | C  (+chorea, global developmental delay, central hypotonia, epilepsy-complex focal seizures) | G | NA | 0 | 3 | 3 | 75.5/75 | 0.66 |
|  |  |  |  |  | Head, eyes, mouth, trunk, left arm, right arm, left leg, right leg |  |  |  |  |  |  |
| Mean/SD |  |  |  |  |  |  | 2.90 ± 3.21 | 11.55 ± 3.91 | 8.65 ± 5.06 | 71.68 ± 26.51/56.43± 32.95 | 23.89 ± 30.59 |

Legend: C, combined dystonia; F, focal dystonia; G, generalized dystonia; H, hemi dystonia; I, isolated dystonia; M, multifocal; NA, information not available; P, phasic; P-T, phasic and tonic; S, segmental; T, tonic; ♂, male; ♀, female.

**Supplementary Table 3**

Stimulation Parameters

| **Patient** | **Electrode Model** | **Right active (negative), positive contacts** | **Voltage/ Frequency/ Pulse Width** | **Left active (negative), positive contacts** | **Voltage/ Frequency/ Pulse Width** |
| --- | --- | --- | --- | --- | --- |
| **1** | Medtronic 3389 | Contact 2 -ve, Case +ve | 2.3 V/ 130 Hz/ 450 μs | Contact 6-ve, Case +ve | 2.1 V/ 130 Hz/ 450 μs |
| **2** | Medtronic 3389 | Contact 0 -ve, Case +ve | 3.4 V/ 130 Hz/ 90 μs | Contact 8 -ve, Case +ve | 3.4 V/ 130 Hz/ 90 μs |
| **3** | Medtronic 3389 | Contact 1 -ve, Case +ve | 1.5 V/ 210 Hz/ 90 μs | Contact 9 -ve, Case +ve | 1.3 V/ 210 Hz/ 90 μs |
| **4** | Medtronic 3389 | Contact 1 -ve, Case +ve | 2.5 V/ 80 Hz/ 60 μs | Contact 9 -ve, Case +ve | 3.1 V/ 80 Hz/ 60 μs |
| **5** | Medtronic 3389 | Contact 1 -ve, Case +ve | 3.6 V/ 130 Hz/ 90 μs | Contact 5 -ve, Case +ve | 5.0 V/ 130 Hz/ 90 μs |
| **6** | Medtronic 3389 | Contact 2 -ve, Case +ve | 3.0 V/ 160 Hz/ 60μs | Contact 10 -ve, Case +ve | 2.5 V/ 160 Hz/ 90μs |
| **7** | Medtronic 3389 | Contact 0 -ve, Case +ve | 4.5 V/ 180 Hz/ 90 μs | Contact 4 -ve, Case +ve | 2.8 V/ 180 Hz/ 90 μs |
| **8** | Boston Scientific Vercise | Contact 2 -ve, Case +ve | 3.0 V/ 130 Hz/ 210 μs | Contact 10 -ve, Case +ve | 4.0 V/ 130 Hz/ 210 μs |
| **9** | Medtronic 3387 | Contact 8, 9 -ve, Case +ve | 2.8 V/ 125 Hz/ 120 μs | Contact 0 -ve, Case +ve | 1 V/ 125 Hz/ 120 μs |
| **10** | Medtronic 3387 | Contact 8, 9 -ve, Case +ve | 9 - 2.8 V/ 145 Hz/ 90 μs | Contact 0, 1 -ve, Case +ve | 2.8 V/ 145 Hz/ 90 μs |
| **11** | Boston Scientific Vercise | Contact 9, 10 -ve, Case +ve | 10 - 2.8 V / 154 Hz/ 90 μs | Contact 1, 2 -ve, Case +ve | 1.5 V / 154 Hz/ 90 μs |
| **12** | Boston Scientific Vercise | Contact 1, 2 -ve, Case +ve | 2.7 V / 130 Hz/ 120 μs | Contact 9, 10 -ve, Case +ve | 2.3 V / 130 Hz/ 120 μs |
| **13** | Boston Scientific Vercise | Contact 9, 10 -ve, Case +ve | 2.4 V / 130 Hz/ 120 μs | Contact 1, 2 | 2.4 V / 130 Hz/ 120 μs |
| **14** | Boston Scientific Vercise Directed | Contact 9 -ve, Case +ve, Contact 10 -ve, Case +ve | 1.5 V/ 130 Hz/ 120 μs | Contact 1 -ve, Case +ve, Contact 2 -ve, Case +ve | 1.5 V/ 130 Hz/ 120 μs |
| **15** | Boston Scientific Vercise Directed | 10, 11, 12, 13, 14, 15 -ve, Case +ve | 3.2 V/ 130 Hz/ 90 μs | Contact 2, 3, 4, 5, 6, 7 -ve, Case +ve | 3.2 V/ 130 Hz/ 90 μs |
| **16** | Medtronic 3387 | Contact 9 -ve, Case +ve | 1.6 V/ 130 Hz/ 60 μs | Contact 0 -ve, Case +ve | 3.4 V/ 130 Hz/ 60 μs |
| **17** | St. Jude Directed 6173 (long) | Contact 10 -ve, Contact 11 +ve. | 4.4 V / 160 Hz/ 100 μs | Contact 1 -ve, Case +ve | 4.0 V / 160 Hz/ 112 μs |
| **18** | Boston Scientific Vercise Directed | Contact 9 (40%) -ve 10-12 (60%) -ve, Case +ve | 2.5 mA / 130 Hz/ 120 μs | Contact 1 (40%), 2-4 (60%) -ve, Case +ve | 2.5 mA / 130 Hz/ 120 μs |
| **19** | Boston Scientific Vercise Directed | Contact 9 (40%) -ve 10-12 (60%) -ve, Case +ve | 2.3 mA / 60 Hz/ 120 μs | Contact 1 (40%), 2-4 (60%) -ve, Case +ve | 2.3 mA / 60 Hz/ 120 μs |
| **20** | Boston Scientific Vercise Directed | Contact 9 (30%) -ve 10-12 (70%) -ve, Case +ve | 1 mA / 130 Hz/ 120 μs | Contact 1 (30%), 2-4 (70%) -ve, Case +ve | 1 mA / 130 Hz/ 120 μs |

Legend: Hz, Hertz; mA, milli Ampere; μs , microsecond; V, Volt.
